# TWEAK Negatively Regulates Human Dicer

**DOI:** 10.3390/ncrna2040012

**Published:** 2016-10-25

**Authors:** Marine Lambert, Geneviève Pépin, Oscar Peralta-Zaragoza, Raphaël Matusiak, Sophia Ly, Patricia Landry, Patrick Provost

**Affiliations:** 1 CHUQ Research Center/CHUL, 2705 Blvd Laurier, Quebec, QC G1V 4G2, Canada; Marine.Lambert@crchudequebec.ulaval.ca (M.L.); genevieve.pepin@hudson.org.au (G.P.); operalta@insp.mx (O.P.-Z.); raphael.matusiak@gmail.com (R.M.); sophia.ly03@gmail.com, (S.L.); patricia.landry@hotmail.com (P.L.); 2 Department of Microbiology-Infectious Disease and Immunity and Faculty of Medicine, Université Laval, Quebec, QC G1V 0A6, Canada

**Keywords:** yeast two-hybrid system, Dicer, TWEAK, protein interaction, microRNA

## Abstract

The ribonuclease Dicer plays a central role in the microRNA pathway by processing microRNA precursors (pre-microRNAs) into microRNAs, a class of 19- to 24-nucleotide non-coding RNAs that regulate expression of ≈60% of the genes in humans. To gain further insights into the function and regulation of Dicer in human cells, we performed a yeast two-hybrid (Y2HB) screen using human Dicer double-stranded RNA-binding domain (dsRBD) as bait. This approach identified tumor necrosis factor (TNF)-like weak inducer of apoptosis (TWEAK) as a Dicer-interacting protein candidate. Confocal immunofluorescence microscopy revealed the colocalization of Dicer and TWEAK proteins at the perinuclear region of HeLa cells. The Dicer-TWEAK protein interaction was confirmed by coimmunoprecipitation and found not likely to be mediated by RNA. TWEAK dose-dependently reduced pre-microRNA conversion into mature microRNA in Dicer activity assays using extracts of transfected human HEK 293 cells. TWEAK expression also impaired microRNA-guided RNA silencing of a reporter gene induced by a pre-microRNA. These findings suggest a role for TWEAK—a pro-inflammatory cytokine—in regulating Dicer function and microRNA biogenesis, and its possible involvement in regulating gene expression during inflammatory processes and diseases.

## 1. Introduction

MicroRNAs are short, 19- to 24-nucleotide (nt) non-coding RNA species that play a vital role in post-transcriptional gene expression, as they modulate ≈60% of the genes in humans [1]. MicroRNAs therefore regulate most biological processes and metabolic pathways, and their deregulation is often associated with genetic diseases, including tumor development and cancer [2].

A core component of the microRNA pathway of human cells is the 217-kDa, microRNA-generating ribonuclease III Dicer [3]. The human Dicer N-terminal ATPase/helicase domain contains a PIWI/Ago/Zwille (PAZ) domain recognizing the 3′ overhang of miRNA precursors (pre-microRNAs), while its C-terminal RNase III domain contains a double-stranded RNA (dsRNA) binding domain (dsRBD) and tandem RNase IIIa/IIIb motifs that cooperate to cleave pre-microRNA substrates into mature microRNAs [4].

Coimmunoprecipitation approaches have been central to identifying Dicer-interacting proteins, such as transactivating response RNA-binding protein (TRBP) [5,6], protein kinase R activator (PACT) [7], and Argonaute 2 (Ago2) [8], which helped elucidate the role and function of Dicer. These studies revealed that TRBP and PACT may act as co-factors of the microRNA processing activity of Dicer [5,6,7], while Dicer interaction with Ago2 may facilitate effector RNA-induced silencing complex (RISC) assembly after microRNA maturation [8]. Investigating the functional relationship between Dicer and downstream effector proteins, our group has reported that human fragile X mental retardation protein (FMRP) can act as a microRNA acceptor protein for the ribonuclease Dicer and facilitate the assembly of microRNAs on specific target RNA sequences [9].

Whereas the structure, enzyme properties and function of Dicer have been well characterized, relatively little is known about how Dicer is regulated. To address that issue, we aimed to expand the repertoire of known Dicer-interacting proteins by employing a protein partner discovery approach: a yeast two-hybrid (Y2HB) screening of cellular protein candidates encoded by a cDNA library. After having localized Dicer to the perinuclear region of cultured human cells, with the endoplasmic reticulum (ER) marker calreticulin [3], a Y2HB screening strategy identified the resident ER protein cytoskeleton-linking ER membrane protein of 63 kDa (CLIMP-63) as a novel Dicer-interacting protein that may help maintain Dicer protein levels in human cells [10].

Compared to conventional coimmunoprecipitation approaches, whose design aims to isolate stable protein complexes, the Y2HB system (and its reliance on reporter gene expression in vivo) has the advantage of identifying novel and unknown candidate proteins interacting with your bait of interest through weak and/or transient interactions that may be of functional/regulatory importance. The subsequent confirmation of Y2HB results by coimmunoprecipitation (e.g., as in the case of the Dicer-CLIMP-63 interaction) validated the usefulness of this experimental design.

To gain further insights into the function and regulation of human Dicer, we focused on its C-terminal catalytic RNase III domain, which contains the tandem RNase IIIa/IIIb motifs and dsRBD, with the aim of identifying cellular proteins interacting with Dicer, and possibly modulating its enzyme activity. We have thus performed a Y2HB screen using human Dicer dsRBD as bait, given that a bait containing the tandem RNase IIIa/IIIb motifs caused auto-activation of the reporter genes and could not be used. This approach allowed the identification of tumor necrosis factor (TNF)-like weak inducer of apoptosis (TWEAK), a 30-kDa pro-inflammatory cytokine [11], as a potential Dicer-interacting protein. Our findings suggest that TWEAK may interact with Dicer and reduce its pre-microRNA processing activity, thereby providing a mechanism by which inflammatory processes may influence microRNA-regulated gene expression.

## 2. Results

### 2.1. TWEAK Interacts with the C-Terminal dsRBD of Human Dicer in Yeast Two-Hybrid Assays

**Figure 1:**
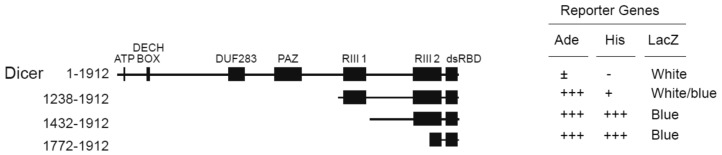
Tumor necrosis factor (TNF)-like weak inducer of apoptosis (TWEAK) interacts with the C-terminal dsRBD of human Dicer. The interaction between TWEAK and truncated forms of human Dicer was tested in a yeast two-hybrid system using strain PJ69-4A. The activity of the adenine (*Ade*), histidine (*His*) and β-galactosidase (*LacZ*) reporter genes was tested to validate the interaction. dsRBD, double-stranded RNA binding domain; DUF283, domain of unknown function 283; PAZ, Piwi-Argonaute-Zwille; RIII, ribonuclease III.

We have performed a Y2HB screen using human Dicer dsRBD (a.a. 1772–1912) [12] as bait, as previously described [13]. Using this approach, we isolated several cDNA clones encoding for TWEAK protein. These clones retested positive for interaction with human Dicer dsRBD in the yeast adenine (*Ade*), histidine (*His*), and β-galactosidase (*LacZ*) reporter gene assays (Figure 1; Dicer a.a. 1772–1912). The TWEAK-Dicer interaction appeared to weaken as the truncation mutants were elongated towards the Dicer N-terminus (Figure 1), possibly because of steric hindrance and/or the misfolding of the fusion protein. These Y2HB results suggest that TWEAK may interact with human Dicer through its C-terminal dsRBD.

### 2.2. TWEAK and Dicer Proteins Colocalize at the Perinuclear Region of HeLa Cells

**Figure 2:**
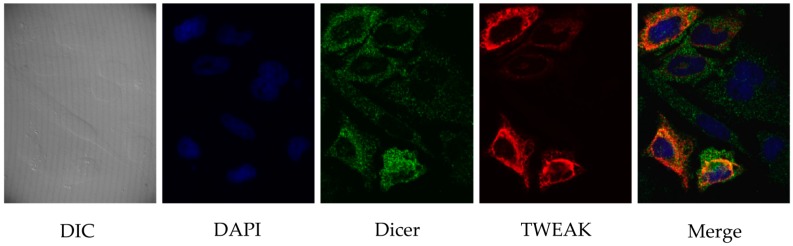
Cytoplasmic/perinuclear colocalization of Dicer and TWEAK proteins in human cells. Flag-TWEAK and human Dicer proteins were expressed in cultured HeLa cells prior to confocal immunofluorescence microscopy. TWEAK was labeled with a rabbit polyclonal anti-FLAG and a secondary anti-rabbit-IgG coupled to AlexaFluor 546 fluorophore (in red), whereas Dicer was labeled using a monoclonal anti-Dicer antibody and a secondary murine anti-IgG coupled to AlexaFluor 488 fluorophore (in green). Cell nuclei were labeled with 4′,6-diamidino-2-phenylindole (DAPI). Proteins were visualized using a confocal microscope (Quorum spinning Disc Wave Fx, Quorum Technologies, Guelph, ON, Canada) and a 63× objective. DIC, differential interference contrast.

To investigate further the relationship between TWEAK and Dicer in human cells, we visualized the intracellular localization of the two proteins in transiently transfected, cultured HeLa cells by confocal immunofluorescence microscopy. As illustrated in Figure 2, Dicer staining was uniformly distributed in the cytoplasm, with a slight enrichment at the periphery of the nucleus (Figure 2, third panel). A faint Dicer signal was also detected in the nucleus. TWEAK showed cytoplasmic/perinuclear localization similar to that of Dicer (Figure 2, fourth panel). Merging of the images produced a yellow staining pattern (Figure 2, last panel) suggestive of a cytoplasmic/perinuclear colocalization of Dicer and TWEAK proteins in cultured human cells.

### 2.3. Dicer and TWEAK Proteins Form a Complex Likely Independent of RNA in HEK 293 Cells

**Figure 3:**
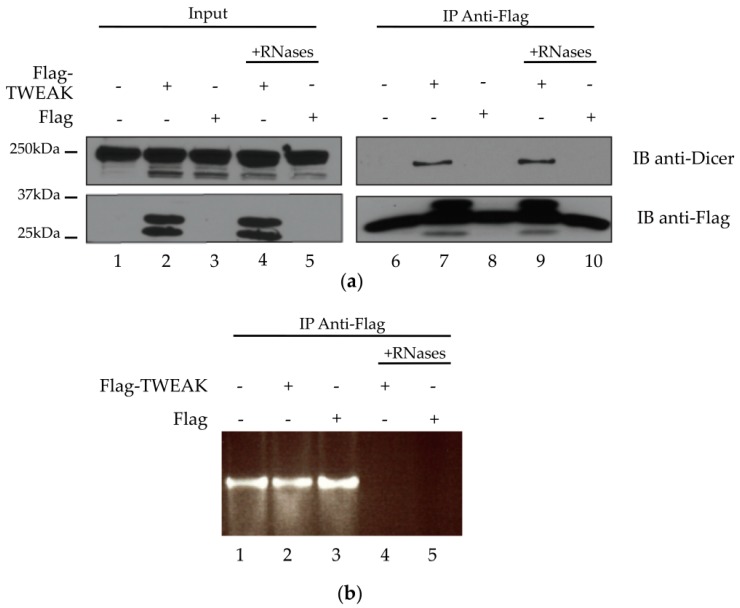
Dicer and TWEAK proteins form a complex likely independent of RNA in HEK 293 cells. HEK 293 cells were transiently transfected with pcDNA3.1-Flag-TWEAK vector, or an empty pcDNA3.1-Flag vector (Flag), prior to Flag-TWEAK protein immunoprecipitation (IP) from untreated lysate (lanes 1–3 and 6–8) or lysate treated with RNases A/T1/V1 (+RNases; lanes 4–5 and 9–10). (**a**) The presence of Flag-TWEAK and Dicer proteins in the lysates (Input) and the IPs was verified by immunoblot (IB) analysis using anti-Flag and anti-Dicer antibodies. The two bands immunoreactive to the anti-Flag antibody likely correspond to different posttranslational forms of the Flag-tagged TWEAK protein. The band across lanes 6 to 10 of the IB anti-Flag panel likely corresponds to the light chain of mouse IgGs; (**b**) RNA degradation upon treatment with RNases A/T1/V1 was confirmed by agarose gel electrophoresis and ethidium bromide staining.

The yeast two-hybrid data and colocalization results prompted us to verify whether TWEAK could interact with Dicer in cultured human cells, by performing coimmunoprecipitation experiments using cleared lysates from transiently transfected HEK 293 cells, as previously described [13]. Dicer (Figure 3a, upper panel, lanes 1 to 5) and Flag-TWEAK (Figure 3a, lower panel, lanes 2 and 4) protein expression was confirmed by immunoblot analysis of the lysates. Flag-TWEAK protein expression did not seem to affect Dicer protein levels (Figure 3a, upper panel), suggesting that TWEAK may not influence Dicer protein synthesis and/or stability.

We were able to immunoprecipitate Flag-TWEAK with a monoclonal anti-Flag antibody (Figure 3a, lower panel, lanes 7 and 9). Immunoblot analysis of the Flag-TWEAK immunoprecipitates, derived from Flag-TWEAK-expressing cells, revealed the presence of Dicer (Figure 3a, upper panel, lane 7 vs. lane 6 or 8). The level of Dicer protein associated with the immunoprecipitates was not affected by pretreatment of the lysates with RNases A/T1/V1 (Figure 3a, upper panel, lane 9 vs. lane 7). Agarose gel electrophoresis and ethidium bromide staining confirmed RNA degradation upon treatment with RNases A/T1/V1 (Figure 3b, lanes 4 and 5 vs. lanes 1–3). Together, these results suggest that TWEAK and Dicer proteins form a complex that is not likely mediated by RNA in human cells.

### 2.4. TWEAK Dose-Dependently Attenuates the Pre-MicroRNA Processing Activity of Dicer

**Figure 4:**
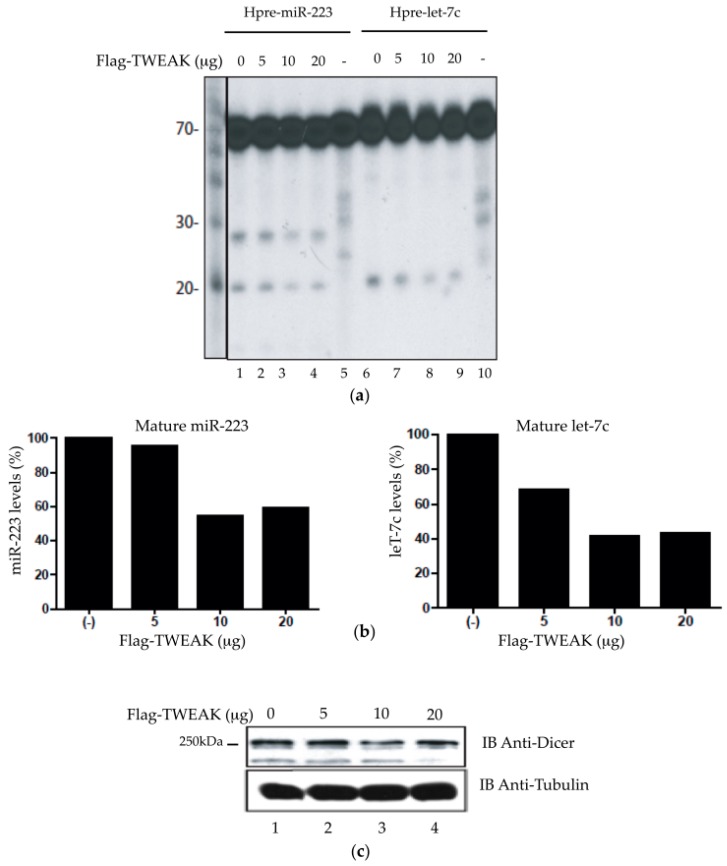
TWEAK dose-dependently reduces pre-microRNA conversion into mature microRNA by Dicer. (**a**,**b**) Cleared lysates (50 µg of proteins) prepared from HEK 293 cells, transfected with increasing amounts of pcDNA3.1-Flag-TWEAK vector (0–20 µg of DNA), were incubated with 5′ ^32^P-labeled human pre-miR-223 (miRBase acc. no. MI0000300) or pre-let-7c (miRBase acc. no. MI0018703). (**a**) The ^32^P-labeled pre-microRNA substrates and cleavage products of Dicer were revealed by denaturing polyacrylamide gel electrophoresis (PAGE)/autoradiography; (**b**) The ≈20-nt bands corresponding to the mature microRNA products were analyzed by densitometry using the ImageJ^®^ software; (**c**) The Dicer protein level in the HEK 293 cell lysates was analyzed by immunoblotting using anti-Dicer antibody, with anti-tubulin as a reference.

Next, we addressed the biological relevance of the TWEAK-Dicer interaction. Considering that TWEAK interacts with Dicer dsRBD, which is located in close proximity to the RNase IIIa and IIIb domains, we reasoned that TWEAK could influence Dicer enzymatic activity. To verify that possibility, we performed an in vitro Dicer activity assay using extracts of transiently transfected HeLa cells [10,12,14]. Addition of the ≈70-nt ^32^P-labeled hsa-pre-miR-223 and hsa-pre-let-7c microRNA substrates led to the formation of their corresponding mature ≈20-nt RNA species (Figure 4a, lanes 1 and 6), which is a signature of the pre-microRNA processing activity of Dicer. Transient Flag-TWEAK expression led to a dose-dependent decrease in the level of mature miR-223 and let-7c microRNA species (Figure 4a, lanes 2 to 4 and lanes 7 to 9), which was confirmed quantitatively (Figure 4b). This effect was not related to changes in Dicer protein levels, as evidenced by anti-Dicer immunoblot analysis (Figure 4c). These results indicate that TWEAK may reduce the ability of Dicer to process their pre-microRNA substrates into mature microRNAs.

### 2.5. TWEAK Impairs MicroRNA-Guided RNA Silencing of a Reporter Gene Induced by a Pre-MicroRNA

The ex vivo microRNA-modulatory effects of TWEAK were corroborated in transiently transfected HEK-293 cells, in which we observed decreased miR-223 levels in cells expressing Flag-TWEAK (Figure S1a) that were not related to changes in Dicer protein expression levels (Figure S1b).

**Figure 5:**
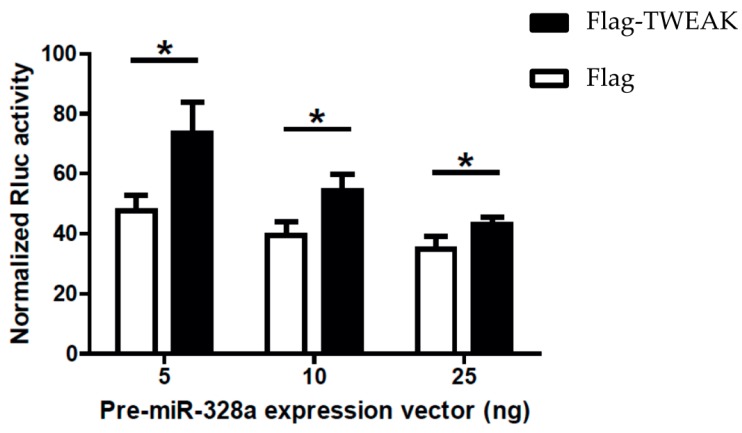
TWEAK impairs microRNA-guided RNA silencing of a reporter gene induced by a pre-microRNA. HEK 293 cells expressing Flag-TWEAK (or transfected with an empty pcDNA3.1-Flag vector; Flag) were transfected with the silencing inducer pre-miR-328a (or a negative pre-microRNA control targeting a deleted region in *Rluc*; shNEG), and a *Rluc* reporter gene [16] coupled with three copies of a natural miR-328a binding site in the 3′ untranslated region (UTR) of the *Rluc* reporter gene. Cells were harvested 18 h later for the successive measurements of Rluc and Fluc activities (*n* = 3 experiments, in duplicate). * *p* < 0.05 versus Flag (empty vector) (Student’s *t* test).

The modulatory effects of TWEAK on the pre-microRNA processing activity of Dicer led us to ask whether TWEAK could influence microRNA-guided RNA silencing. To answer this question, we employed a dual-luciferase reporter gene activity assay in transiently transfected HEK 293 cells, in which silencing of *Rluc* gene expression is induced by a pre-microRNA substrate, as previously described [15]. Expression of pre-miR-328a in mock-transfected cells induced silencing of a *Rluc* reporter gene coupled with three copies of a natural binding site for miR-328a (Figure 5, Flag data), as reported previously [10,16,17,18]. Co-expression of Flag-TWEAK slightly impaired *Rluc* reporter gene silencing induced by pre-miR-328a (Figure 5, Flag-TWEAK vs. Flag data).

Altogether, these results support a role for TWEAK in regulating the efficiency of the microRNA pathway through interaction with Dicer and modulation of its pre-microRNA processing actvity in human cells.

## 3. Discussion

The biogenesis and function of microRNAs is relatively well known, but the mechanisms underlying regulation of the microRNA pathway itself remain more elusive. Here, the use of a yeast two-hybrid screening approach allowed the identification of TWEAK as a new protein interactor and regulator of the microRNA-generating enzyme Dicer, which may modulate the efficiency of the microRNA pathway in human cells. 

Identified as a potential Dicer-interacting protein by using human Dicer C-terminal dsRBD as bait, TWEAK did not interact with full-length, 218-kDa human Dicer in the yeast two-hybrid system. Such a discrepancy may be observed with large proteins, in which some domain structures may occlude, mask, or block access to other regions of the protein; this represents a limitation inherent to this technique [15]. Interestingly, Dicer ribonuclease activity is self-inhibited by its N-terminal helicase domain, which can be relieved by its cofactor TRBP, probably through a conformational rearrangement that limits self-inhibition [17]. In fact, two Dicer-interacting proteins, TRBP and PACT, interact with the helicase domain of Dicer and induce a change in its conformation [18]. Knowing that TWEAK interacts with Dicer via its dsRBD, which is in close proximity to the RNase III domain tandem, these observations provide a rational basis to explain the loss of TWEAK-Dicer interaction upon elongation of the truncation mutants towards the Dicer N-terminus or when studying the full-length Dicer protein; hence, the need to confirm the Dicer-TWEAK interaction in vivo.

The fact that TWEAK may not interact with the full-length human Dicer protein, in yeast cells, suggests that this interaction may require, in human cells, posttranslational modification(s) induced upon specific conditions or another protein partner that would either make the Dicer dsRBD accessible to TWEAK or serve to adjoin the two proteins together. Considering that the accessibility of the catalytic, C-terminal RNase III domain tandem of Dicer may be hampered by its N-terminus, it may be speculated that a protein that binds to the N-terminal helicase domain of Dicer, like TRBP or PACT, may play such a role. Another possible candidate is CLIMP-63, a resident endoplasmic reticulum protein that (i) interacts with Dicer via its N-terminal protein interaction domain (PID; amino acids 242–430) [19,20], and (ii) exhibits an intracellular localization similar to that of TWEAK [10].

The Dicer-TWEAK interaction may also be modulated by cellular proteins interacting with the Dicer dsRBD, such as the 5-lipoxygenase (5LO) enzyme. We have previously reported that 5LO could interact with the C-terminal domain of Dicer and modify its pre-microRNA processing activity [12]. In return, Dicer increased 5LO enzyme activity. Notably, TWEAK and 5LO share the same Dicer-interacting domain (i.e., dsRBD), and both are important mediators of inflammation. Indeed, differentiated immune cells (e.g., granulocytes, lymphocytes, and monocytes) express TWEAK and 5LO, suggesting a link between inflammation and regulation of gene expression by microRNAs.

The function of the Dicer dsRBD is to stabilize the binding interactions between the ribonuclease and pre-microRNAs [12]. The observed decrease in pre-microRNA processing activity of human Dicer induced by TWEAK may thus imply an interference with Dicer dsRBD function caused by TWEAK binding. This scenario is not incompatible with, and is rather supported by, the Dicer-TWEAK interaction not involving single-stranded or double-stranded RNA (like that between Dicer and CLIMP-63 [21]), as Dicer-TWEAK complexes would form prior to pre-microRNA binding and processing. This is in contrast with heterogeneous nuclear ribonucleoprotein A1 (hnRNP A1) [22] or KH-type splicing regulatory protein (KSRP) [23] proteins, which modulate pre-microRNA processing by interacting with their specific loop sequences rather than directly with Dicer.

Our results indicate that TWEAK binds to Dicer, interferes with its capacity for microRNA biogenesis and impairs the efficiency of the microRNA pathway of human cells, possibly modulating the level and gene-regulatory effects of microRNAs. Interestingly, Panguluri et al. observed a decrease in the level of most microRNAs and changes in the expression of several mRNAs when studying the effects of TWEAK expression on the transcriptome profile of myotubes [22,23]. Could these effects of TWEAK on the microRNA and mRNA transcriptome be mediated, at least to some extent, through its interaction with Dicer? To answer this question, however, we have to consider that TWEAK is a multifunctional cytokine that binds to fibroblast growth factor-inducible 14 (Fn14) receptor to activate several signalling pathways, such as the NF-κB pathway [24], which, by themselves, may influence the expression of several genes.

The existence of two isoforms of the TWEAK protein adds another layer of complexity. A full-length transmembrane TWEAK protein, expressed in leukocytes (e.g., granulocytes, lymphocytes, and monocytes) [25,26], can be cleaved by a furin to release an extracellular, shorter form of soluble TWEAK, defined as sTWEAK [27]. A Dicer-TWEAK interaction may thus occur within a TWEAK-expressing cell or in a cell internalizing sTWEAK released by another cell. In TWEAK-expressing cells, Dicer may interact with full-length TWEAK protein at the endoplasmic reticulum. Upon inflammation, cleavage and secretion of TWEAK would enhance the availability of Dicer for microRNA biogenesis, thereby enabling changes in gene expression in response to inflammation. On the other hand, Dicer may interact with sTWEAK internalized by the cells exposed to the cytokine, as a result of inflammation [11]. Primary macrophages exposed to sTWEAK readily internalize this cytokine, although the mechanism that mediates the cellular uptake of TWEAK has not been identified [28]. If sTWEAK is internalized by the scavenger receptor CD163, it would be delivered to lysosomes, which greatly limits the possibility of an interaction with Dicer, although mechanisms of lysosomal escape exist [29,30]. Intriguingly, several cell types that lack the two known receptors for TWEAK, namely Fn-14 and CD163, are still capable of internalizing sTWEAK [25,31], suggesting the existence of alternate uptake mechanism(s).

Recently, plasma membrane receptor Death receptor 5 (DR5), also known as TRAIL receptor 2 (TRAILR2), has been reported to interact with the Microprocessor complex in the nucleus and to inhibit microRNA biogenesis in cancer cells [26]. Given that TRAIL-R2 and TWEAK are both members of the tumor necrosis factor receptor family and plasma membrane proteins [32], it would be tempting to speculate that TWEAK may interact with Dicer and modulate microRNA biogenesis in the cytoplasm the same way as TRAIL-R2 does with the Microprocessor complex in the nucleus.

The findings that we have obtained, based on TWEAK overexpression, suggest that TWEAK negatively regulates the pre-microRNA processing activity of Dicer, reduces mature microRNA levels, and impairs microRNA-guided RNA silencing. We have no reason to believe that knockdown of TWEAK expression, in TWEAK-expressing cells, would not produce the opposite effects of TWEAK overexpression, i.e., upregulation of pre-microRNA processing activity of Dicer, increase of mature microRNA levels, and amelioration of microRNA-guided RNA silencing in human cells.

In conclusion, the results of this study support a role for TWEAK as a new regulator of the microRNA processing activity of the ribonuclease Dicer and of the microRNA pathway of human cells, thereby expanding the role and function of this cytokine. The effects of TWEAK on the cellular programming of gene expression are likely to be multifactorial and may underlie the alterations in cellular processes—such as cell proliferation, apoptosis, and angiogenesis—observed in inflammatory and autoimmune diseases, as well as in cancer, which may well justify the use of TWEAK as a biomarker of these pathological conditions [33]. Further investigations on the role and importance of TWEAK, including sTWEAK, in microRNA-mediated regulation of gene expression, and of the underlying mechanisms, may bring new insights into the etiology and pathogenesis of inflammatory and autoimmune diseases.

## 4. Materials and Methods

### 4.1. Plasmid DNA Constructs

Human Dicer was amplified by polymerase chain reaction (PCR) and cloned into a modified mammalian expression vector pcDNA3.1 (Thermo Fisher Scientific, Waltham, MA, USA) containing an N-terminal FLAG epitope (pcDNA3.1.-5′Flag), as described previously [34]. Human Dicer open reading frame and various deletion mutants of Dicer were amplified by PCR and cloned in frame into the BamHI/SalI sites of pGBT9 (Clontech, Mountain View, CA, USA) or the EcoRI/XhoI sites of pACT2 (Clontech). TWEAK cDNA was amplified by PCR for cloning into pGBT9, pACT2, pcDNA3.1 (Invitrogen), and pcDNA3.1-5′Flag vectors. Yeast two-hybrid strain (PJ69-4A), vectors (pGBT9, pGBT9-SNF1, pACT2-SNF4), and reporter gene assays were described previously [3]. The known two-hybrid interactors SNF1 and SNF4 were used as a positive control [14]. The dual-luciferase reporter construct psiCHECK-miR-328a 3xBS has been described previously [13]. The integrity of the constructs was verified by DNA sequencing.

### 4.2. Yeast Two-Hybrid Experiments

Yeast strain PJ69-4A was used for the two-hybrid cDNA library screening and assays [15,16], as described in Provost et al. [17]. Yeast PJ69-4A harboring the pGBT9-Dicer vector was transformed with a human lung cDNA library, and growers on synthetic dropout (SD)/-Leu/-Trp/-Ade plates were tested for activation of their adenine, histidine, and lacZ reporter genes. The interacting pACT2 plasmid was rescued from positive clones and retested to confirm the interaction prior to sequencing of the interacting cDNA inserts. For yeast two-hybrid assays, PJ69-4A cells were co-transformed with various pGBT9 and pACT2 constructs and tested for reporter gene activation six days later, as described previously [13].

### 4.3. Confocal Immunofluorescence Microscopy

Double immunofluorescence staining was performed on HeLa cells grown on sterile glass coverslips coated with poly-l-lysine in Dulbecco’s minimal essential medium supplemented with 10% (*v*/*v*) fetal bovine serum (FBS), 1 mM sodium pyruvate, 100 units/mL penicillin and 100 μg/mL streptomycin in a humidified incubator under 5% CO_2_ at 37 °C. Cells were transfected with plasmid constructs encoding epitope-tagged TWEAK and Dicer proteins using Lipofectamine 2000 (Invitrogen) and harvested 20 h post-transfection, as described previously [14]. Cells were washed in phosphate-buffered saline (PBS), fixed in 4% (*w*/*v*) paraformaldehyde, permeabilized with 0.1% Triton X-100, and incubated in blocking buffer (PBS containing 10% FBS). Cells were then incubated with rabbit polyclonal anti-Flag (dilution 1/500; Santa Cruz Biotechnologies, Inc., Dallas, TX, USA) and mouse monoclonal anti-Dicer (dilution 1/1000; Abcam, Toronto, ON, Canada) antibodies, respectively. After extensive washing in PBS, the cells were incubated with Alexa Fluor 488 (green)-conjugated goat anti-mouse or Alexa Fluor 546 (red)-conjugated goat anti-rabbit secondary antibodies (dilutions 1/500; Molecular Probes, Eugene, OR, USA). Cell nuclei were labeled with DAPI (4′,6-diamidino-2-phenylindole). After extensive washing in PBS, the coverslips were mounted on slides with Prolong Gold anti fade reagent (Molecular Probes). Labeling of HeLa cells was visualized with an inverted Olympus IX70 microscope (90× magnification), and the images prepared with Image J 1.38x software (https://imagej.nih.gov/ij/).

### 4.4. Protein Extracts and Western Blot Analysis

Proteins were extracted by harvesting cells in lysis buffer (50 mM Tris–HCl, 137 mM NaCl, 1% Triton X-100, 1 mM phenylmethylsulfonyl fluoride (PMSF), 1× protease inhibitor cocktail mix without ethylenediaminetetraacetic acid (EDTA), pH 8.0) [14] and incubating the lysates for 15 min on ice. The lysates were clarified by centrifugation at 16,000× *g* for 15 min at 4 °C. Protein concentration of the cleared lysates was determined by the method of Bradford using the Bio-Rad (Hercules, CA, USA) dye reagent, with bovine serum albumin as standard. Protein extracts were analyzed by Western blot using mouse monoclonal anti-Dicer (Abcam) [16] and rabbit polyclonal anti-Flag M2 (Sigma, St. Louis, MO, USA) primary antibodies.

### 4.5. Immunoprecipitation Experiments

HEK 293 cells were transiently transfected with plasmid constructs encoding epitope-tagged TWEAK protein or the empty vector (mock) by the calcium phosphate method and harvested 48 h later. Cells were washed twice with ice-cold PBS and solubilized with 1 mL of lysis buffer (50 mM Tris–HCl, 137 mM NaCl, 1% Triton X-100, 1 mM PMSF, pH 8.0, supplemented with complete protease inhibitor cocktail (Roche, Mississauga, ON, Canada). The lysate was kept on ice for 15 min, clarified by centrifugation at 13,000× g for 1 min, and the supernatant was preserved. An aliquot of the supernatant was kept to determine protein concentration by Bradford and analyze TWEAK and Dicer protein expression by immunoblotting. In order to confirm that the protein interaction under study does not involve RNA, we incubated the lysates with a cocktail of ribonucleases (RNases A/T1/V1) prior to immunoprecipitation, as described previously [10,35]. Cleared lysates (2 mg proteins) were incubated with 1 μg of anti-Flag M2 antibody (Sigma, St. Louis, MO, USA) for 1 h at 4 °C under continuous rotation to immunoprecipitate Flag-TWEAK protein, as described previously [10]. Then, pre-washed Dynabeads® Protein G beads (≈10 µL packed beads; Thermo Fisher Scientific) were added, and the incubations continued for an additional hour. The beads were then washed four times with lysis buffer prior to adding loading buffer and boiling for 5 min to elute the immune complexes. The immunoprecipitated proteins were electrophoresed on a 7% sodium dodecyl sulfate-polyacrylamide gel electrophoresis (SDS-PAGE), transferred to a polyvinylidene difluoride (PVDF) membrane and immunoblotted for the presence of TWEAK or Dicer, with an anti-Flag or anti-Dicer antibody, respectively.

### 4.6. Dicer Activity Assays

Recombinant human Dicer full-length protein was incubated in the absence or presence of TWEAK protein during 30 min prior to addition of a randomly, ^32^P-labeled microRNA precursor substrate (pre-let-7a-3) in a reaction buffer containing 20 mM Tris–HCl, 5 mM MgCl_2_, 1 mM dithiothreitol (DTT), 1 mM ATP, 5% Superase•In (Ambion, Carlsbad, CA, USA), pH 7.5, at 37 °C for 1 h. The resulting RNA products were analyzed by denaturing PAGE and autoradiography, and the intensity of the bands was quantitated by densitometry, as described previously [35].

### 4.7. Reporter Gene Activity Assay

HEK 293 cells grown in 6-well plates were transfected with psiCHECK-miR328a 3xBS reporter construct (100 µg DNA) without (mock; empty pcDNA3.1-Flag vector) or with pcDNA3.1-5′Flag-TWEAK, and the silencing inducer pre-miR-328a (Promega, Madison, WI, USA) or its negative pre-microRNA control targeting a deleted region in *Rluc* (shNEG; Promega) (5, 10 or 25 ng RNA). Eighteen hours later, cells were harvested, and Renilla luciferase (Rluc) and Firefly luciferase (Fluc) activities were measured, as described previously [35]. Results of *Rluc* activity were normalized with Fluc reporter activity, and expressed as percentage of the results obtained from the shNEG control. A Student’s *t* test was performed to evaluate the effect of TWEAK, with *p* value <0.05 considered significant.

## Supplementary Materials

The following are available online at www.mdpi.com/2311-553X/2/4/12/s1.
