# Supplementary material for: TWEAK Negatively Regulates Human Dicer
Source: Noncoding RNA. 2016 Oct 25;2(4):12. doi: 10.3390/ncrna2040012 (PMC5831928; doi:10.3390/ncrna2040012)
Supplement: Supplementary file 1 [file ncrna-02-00012-s001.pdf]

Supplementary Materials: TWEAK Negatively Regulates Human Dicer

Marine Lambert, Geneviève Pépin, Oscar Peralta-Zaragoza, Raphaël Matusiak, Sophia Ly, Patricia Landry and Patrick Provost

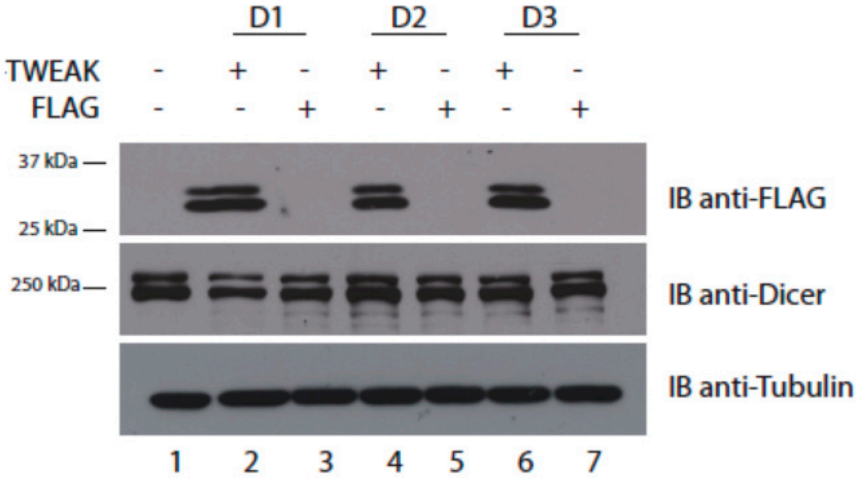

(**a**)

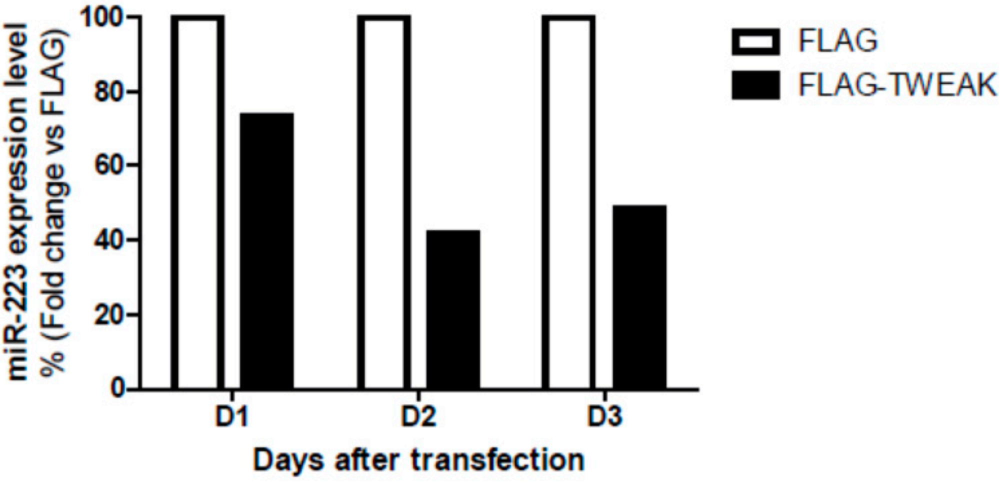

(**b**)

Figure S1. TWEAK may reduce microRNA expression levels in human cells. (**a**,**b**) HEK 293 cells transiently transfected with Flag-TWEAK or an empty pcDNA3.1-Flag vector (Flag) and a vector expressing the precursor of the miR-223 were harvested 1 (D1), 2 (D2), or 3 (D3) days after transfection. Total RNA was extracted with TRIzol® (Qiagen, Toronto, ON, Canada), and the samples were analyzed by quantitative PCR (qPCR) to measure the level of mature miR-223. Results were normalized by the $2^{-\Delta\Delta Ct}$ method using the RNU6 housekeeping control, and expressed as a percentage. The experiment was repeated with cells extracted in T8 buffer to harvest proteins. The protein extracts (50 μg) were analyzed by 7% SDS-PAGE, transferred on a PVDF membrane and immunoblotted with anti-Dicer (Abcam, 1/5000), anti-TWEAK (Cell Signaling, Danvers, MA, USA, 1/5000), and anti-tubulin (DSHB, North Liberty, IA, USA, 1/10,000), used as a loading control. Bands were visualized by chemiluminescence.
